# Does Economic Support Have an Impact on the Health Status of Elderly Patients With Chronic Diseases in China? - Based on CHARLS (2018) Data Research

**DOI:** 10.3389/fpubh.2021.658830

**Published:** 2021-04-20

**Authors:** Shaoliang Tang, Yun Xu, Zhengjun Li, Tongling Yang, David Qian

**Affiliations:** ^1^School of Health Economics and Management, Nanjing University of Chinese Medicine, Nanjing, China; ^2^Swinburne Business School, Swinburne University of Technology, Melbourne, VIC, Australia

**Keywords:** elderly patients, chronic disease, health status, regional differences, economic support

## Abstract

**Objective:** The economic support of elderly patients with chronic diseases may affect their health status. This study assessed the impact of economic support on the health status of elderly patients with chronic diseases in China and explored the relationship between regional economic differences and the impact of economic support on health status.

**Methods:** This study used the data of 3,416 elderly patients with chronic diseases from the China Health and Retirement Longitudinal Study (CHARLS) in 2018. Logit model and stepwise regression method were used to analyze and compare the influence of economic support on self-rated health, mental health, and activities of daily living (ADL) of elderly patients with chronic diseases. Sub-regional comparison was used for the research of regional differences in the impact of economic support on health.

**Results:** we find that economic support has a positive effect on the health status of elderly patients with chronic diseases in China. Socioeconomic support has a positive effect on self-rated health and mental health of elderly patients with chronic diseases, and the effect on activities of daily living (ADL) is no longer significant after the gradual inclusion of control variables. Family economic support has no significant impact on the health dimensions of elderly patients with chronic diseases. With respect to regional differences, socioeconomic support can improve mental health in central China and improve self-rated health in western China, while it can improve overall health status in eastern China. The influence of family economic support on different health dimensions in different regions is still insignificant.

**Conclusions:** The health status of elderly patients with chronic diseases is affected by socioeconomic support in China. In order to improve the health of elderly patients with chronic diseases, it is necessary to increase socioeconomic support. Meanwhile, it is also essential to pay attention to the differences in economic support between different regions and increase the socioeconomic support for elderly patients with chronic diseases in undeveloped regions so as to improve their health status.

## Introduction

China's rapid economic development has brought both improvements in living standards and medical technology since the 21st century. Life expectancy has risen significantly, but so has the proportion of the elderly in the population. Compared with other countries, China's aging is characterized by high degree and rapid progress (1–3). Meanwhile, China's spectrum of disease is changing against the backdrop of aging and a dramatic rise in life expectancy (4). Infectious diseases have been effectively controlled, the maternal mortality rate and the population mortality rate have been significantly reduced (5). However, the number of patients with chronic diseases is increasing, showing an upward trend year by year. It is expected to increase by 50% by 2030 without effective control measures (6–8). According to data from the China Health Statistics Yearbook, the prevalence of chronic diseases among residents over 65 years old in the regions surveyed in 2018 was as high as 62.3%. It shows a trend that the prevalence of chronic diseases in the elderly is high and chronic diseases are more common in the elderly (9).

Chronic diseases have the characteristics of high morbidity, low cure rate, and high mortality. Many chronic diseases require long-term care and are lifelong diseases, which directly lead to the continuous medical expenditure of patients (10, 11). Chronic diseases of the elderly also present the characteristics of high disability rate and multiple diseases. Diseases affect each other, and the group of elderly suffering from two or more chronic diseases is gradually expanding (12–14). Numerous studies have also shown that the chronic diseases not only bring damage to the physical health of the elderly, but also affect the mental health of elderly patients with chronic diseases (15, 16). Some scholars have pointed out that elderly patients with chronic diseases will suffer psychological burden due to the continuous economic expenditure brought by chronic diseases (17, 18). Therefore, chronic diseases have become an important factor affecting the health-related quality of life of most elderly people (19–21).

The World Health Organization (WHO) estimates that more than two thirds of all deaths worldwide are caused by chronic diseases, and these diseases are usually related to the elderly. It reveals that economic conditions are closely linked to chronic diseases. Because of the refractory characteristics of chronic diseases, the medical treatment of elderly patients with chronic diseases needs long-term economic support. Christy Pu states that economic conditions have a significant impact on the health of the elderly (22). However, among the elderly in China, the number of people with earned income is relatively small. Family pension is the most common source, and the elderly needs to rely on family economic support when there is no or insufficient pension (23). Under the circumstance of low-income, the cost of health care for chronic diseases quickly drains household savings and pushes millions into poverty each year (24).

Most existing studies have focused on the impact of economic support on the health of the elderly or patients with chronic diseases, only a few studies combine the elderly and patients with chronic diseases. Elderly patients with chronic diseases are different from others in that most of them have no income from work and need to rely on pension or long-term family economic support to treat chronic diseases. With respect to economic support, most of the studies only consider a single type of economic support as factor which affects health effect. The economic support stipulated in this paper combines earned income, pension, and family economic support, could better represent the economic conditions in elderly patients with chronic diseases.

Three dimensions of health including self-rated health, mental health, and activity of daily life (ADL) are used to measure health status. Self-rated health is one of the most common indicators used by various databases and scholars to measure the status of individual health, which could effectively represent the status of individual health (25). Studies also have shown that self-rated health can accurately assess the physical and mental health (19, 26). Chronic diseases will make elderly patients suffer from psychological burden as well (27). Therefore, mental health is applied in this paper. Self-rated health and mental health are both based on patients' subjective judgment and are vulnerable to interference. An objective indicator activity of daily life (ADL) is also included in this paper to measure the health status by inquiring the respondents whether they have the basic ability to care for their own needs independently.

Considering the large regional economic disparity, this paper divides China into eastern, central, and western regions based on the geographic location. We study the economic support of elderly patients with chronic diseases in three regions respectively, to measure whether there are regional differences in the influence of economic support on different health dimensions of elderly patients with chronic diseases.

On this basis, this paper uses data of China Health and Retirement Longitudinal Study (CHARLS) conducted by Peking University in 2018, taking elderly patients with chronic diseases aged 60 and above as the research object, examining the impact of economic support on the health status of elderly patients with chronic diseases in China.

## Methods

### Study Design and Sample Selection

Based on the China Health and Retirement Longitudinal Study (CHARLS) database, this paper studied the impact of economic support on the health status of elderly patients with chronic diseases in China. The China Health and Retirement Longitudinal Study (CHARLS) national baseline was launched in 2011 and the data is tracked every 2–3 years. CHARLS has more than 17,000 respondents who are from various regions in China, covering 150 county-level units and 450 village-level units. When sampling, CHARLS uses PPS (Probability Proportional to Size) to select county level units first and then selects village or community level units. In each village or community, CHARLS randomly selects 25–36 residences from the map, and determines the number of samples of family households in each residence. Besides, there is a section of filter at the beginning of the CHARLS questionnaire which can eliminate invalid questionnaires. Therefore, the data can well-represented the overall situation of China and the quality of data is guaranteed.

CHARLS includes basic information, family, health status and function, cognition and depression, health care and insurance, work and retirement, pensions, income expenditure and assets, real estate, and housing, with a wide range of information. It is a reliable database for studying health levels and influencing factors in middle-aged and elderly people.

Patients aged 60 and above with chronic diseases were selected as the research objects in this paper. After eliminating the samples lacking key variables, 3,416 samples were finally utilized. The database used in this paper is made available to the academic community with the approval of the Ethics Committee of Peking University, so ethical approval is not required.

### Variable Description

The economic support of the elderly patients with chronic diseases studied in this paper consists of two parts, namely family economic support within 1 year and socioeconomic support within 1 year. Among them, family economic support includes those provided by children, parents and siblings. Socioeconomic support includes pensions and earned income of reemployment. Pensions refer to income of government organization and institution, enterprise worker primary endowment insurance, endowment insurance of urban and rural dweller, commercial endowment insurance, and other endowment insurance. Earned income refers to the salary received from work, including bonuses and various subsidies.

Self-rated health is based on the respondent's self-rating of current status of health, reflecting the respondents' assessment of their own health. Self-rated health in the CHARLS questionnaire is achieved by asking patients “How would you describe your health condition?” to measure. The question is divided into five levels, which are “very good,” “good,” “common,” “poor,” and “very poor.” According to the results of the questionnaire, the OLS regression and ordered logit regression have been carried out, respectively. Although, same conclusions are obtained by the OLS regression and ordered logit regression, logit regression can judge the health status more directly. Meanwhile, ordered logit regression can eliminate the influence of threshold of dichotomous dependent variable on the significance of the estimated coefficient. All the statistical procedures in this article are implemented by STATA15.

A large number of studies have shown that depression is the most common mental health problem of the elder, which is representative of the individual's mental health status. Therefore, in this paper, the levels of mental health are measured through the depression status of the respondents (28). CHARLS used a 10-item central depression scale modified by Andresen in 1994. Due to its good reliability and validity, short response time, and high recovery rate, this scale has great potential to be applied in large-scale investigation and study (29), it is considered that this scale can effectively measure the depression level of the elderly in CHARLS data. The scale is composed of 10 symptoms description and respondents are asked to answer the frequency of described symptoms in last week. The symptom descriptions including “I was bothered by things that don't usually bother me,” “I had trouble keeping my mind on what I was doing,” “I felt depressed” “I felt everything was difficult,” “I felt hopeful about the future,” “I felt fearful,” “I could not sleep well,” “I was happy,” “I felt lonely,” “I found hard to keep going.” The respond options include: “Rarely or none,” “unusual,” “sometimes or half of the time,” and “most of the time.” The result split into/falls into four categories. The four level are counted as 0, 1, 2, and 3 points. There are two questions with positive emotion options for reverse scoring, namely 3, 2, 1, and 0 points. The total score of the 10 items in this scale is between 0 and 30 points. It is considered that ≥10 points can be considered as mild depression, and ≥15 is considered as high depression. In this paper, according to the general standard, a score of <10 is assigned to a value of 0 to represent non-depression, while a score of 10–15 is assigned to 1 to represent mild depression and a score of ≥15 is assigned to 2 to represent severe depression.

Good functional status is the basis for maintaining the independence of the elderly. The elderly often suffer from a variety of chronic diseases, which cause health damage. The more types of chronic diseases, the greater risk of disability, which can lead to decreased ability of daily living (ADL) (30). ADL is an important indicator to measure the health status of the elderly, and its evaluation can provide a basis for the diagnosis of diseases, the prediction of social service needs of the elderly, the formulation of treatment plans, and the reasonable placement of the elderly (31). The ADL scale in the CHARLS questionnaire includes two parts: physical life scale (ADL) and instrumental daily life self-care scale (IADL). Since ADL stands for basic ability to act and can better reflect the health status of the respondents. This paper chooses the physical life scale (ADL) as the evaluation criteria that whether the respondents have difficulties in dressing, bathing, eating, getting up, using the toilet, controlling urination, and defecation. The respondents will be marked as‘1' if they meet any criterion above and marked as ‘0' if none of symptom happened.

The control variables are divided into four categories: demographic characteristics, unhealthy lifestyles (drinking and smoking), physical activities, and medical insurance. Demographic characteristics include gender, age, marital status, education level, type of residence. Where in Marital status is reclassified according to the six options in the questionnaire. The six options are “married and living with a spouse,” “married but temporarily not living with the spouse for work and other reasons,” “separated, no longer living together as a spouse,” “divorced,” “widowed,” and “never married,” the first three options are reclassified into “married” and the last are reclassified into “single.” Previous studies (Erpeng Liu) have pointed out that smoking and drinking will have an impact on health as unhealthy lifestyles (32). Meanwhile, physical exercise is also one of the factors affecting health. Narimasa Kumagai's research further shows that poor health behaviors have negative effect on daily physical activities, while increasing the intensity of daily physical activities has a positive impact on individual health status (33). Therefore, we regard “Whether Smoke” and “Drinking Frequency” as one type of control variable (unhealthy lifestyles), and “Physical Activity Intensity” as another type of control variable. Zeng Yanbing's research shows that the type of medical insurance will affect whether the elderly seek medical treatment or not, and the medical insurance for urban workers will improve the utilization rate of outpatient services for the elderly (34). Therefore, this paper regards the type of medical insurance as control variable. The insurance type question corresponds to the questionnaire's EA001, in which CHARLS divides the medical insurance into 10 categories. This paper categorized the insurance with insignificant number of participants as “Other Insurance,” (amongst are Government Medical Insurance, Medical Aid, Private Medical Insurance: Purchased by Work Unit, Private Medical Insurance: Purchased by Individual, Urban Non-Employed Person's Health Insurance, long-term Care Insurance). As result, this paper identified five medical insurance categories: Urban employee medical insurance, Urban, and rural resident medical insurance, Urban resident medical insurance, New rural cooperative medical insurance, and other insurance. At the same time, the medical insurance reimbursement expense of the sample population is also recognized as a control variable due to its significance as an economic indicator related to health. The description and assignment of variables are shown in Table 1.

**Table 1:** Variable description.

**Variable**	**Description of variable setting**
SRH	Very poor = 0
	Poor = 1
	Normal = 2
	Good = 3
	Very good = 4
Depression	Non-depression = 0
	Mild depression = 1
	Severe depression = 2
ADL	No difficulty = 0
	Difficulty = 1
Gender	Male = 1
	Female = 2
Marital status	Single = 1
	Married = 2
Education	Primary school or below = 1
	Junior high school = 2
	Senior high school = 3
	College or above = 4
Residence	Village = 1
	Combination zone between urban and rural areas = 2
	The center of city/town = 3
Activity	Mild activity = 0
	Moderate activity = 1
	Vigorous-intensity activity = 2
Smoke	No = 0
	Yes = 1
Drink	Drink more than once a month = 1
	Drink but less than once a month = 2
	None of these = 0
Types of medical insurance	Urban employee medical insurance = 1
	Urban and rural resident insurance = 2
	Urban resident medical insurance = 3
	New rural cooperative medical insurance = 4
	Other = 5
Region	East = 1
	Central = 2
	West = 3

### Statistical Analysis

Since ADL has only two dimensions, namely healthy and unhealthy, this paper chooses Logit model to study the impact of economic indicators on ADL, and sets the model as follows:

(1) $$H_{i}=\alpha +\beta S_{i}+\gamma X_{i}+\varepsilon$$

Here, *H*_*i*_ represents the status of ADL. *S*_*i*_ is the key independent variable, representing the economic support the respondents received. *X*_*i*_ represents control variables, including demographic indicators (gender, age, marital status, education level, type of residence), health behavior (physical activity intensity, whether smoking, and drinking frequency), the type of medical insurance, and medical insurance reimbursement expense.

In this paper, the measurement of self-rated health and mental health is ordered multiple classification. We choose ordered Logit model to carry out regression analysis.

(2) $$H_{i}*=\alpha X_{i}+\varepsilon_{i},\;i=1,2,3(4,5)$$

(3) $$H_{i}=\;\left\{ \begin{array}{l} \;1,\;H_{i}^{*}\leq \;\beta_{1}\\ 2,\;\beta_{1}\leq H_{i}^{*}\leq \;\beta_{2}\\ 3,\;\beta_{2}\leq H_{i}^{*}\leq \;\beta_{3} \end{array}\right.$$

Here, ${\text{H}}_{\text{i}}^{*}$ is the latent variable, cannot observe the specific value. **H**_**i**_ represents the status of health (SRH, Depression). **X**_**i**_ represents control variables, including economic support, demographic indicators (gender, age, marital status, education level, type of residence), the type of medical insurance, and medical insurance reimbursement expense. **i** represents the observed value, α represents the value of the parameter variable to be estimated. ***ε*** is the random disturbance term, following the Logistic distribution. ***β*** is the boundary point of the interval.

## Results

### Basic Characteristics of Elderly Patients With Chronic Diseases

Table 2 shows the basic characteristics of the investigated patients with chronic diseases. Overall, women make up the majority of the respondents, accounting for 70% of the population. More than half of the population is between 60 and 70 years old, and people over 80 years old account for more than 10% of the total population, indicating that China's overall health is in a good status. The married rate is 70%, because the widowhood rate and divorce rate of the elderly group are higher than that of the general population, so they are more likely to be single. Similar to this is the level of education. Due to the low education level in China before the 1970's, more than 90% of the elderly have an education level below junior high school. It is worth noting that the proportion of the elderly with an education level below primary school level in western China is significantly higher than that in eastern and central China, which indicates the exists of certain level of educational inequality in China. From the point of residence, most of the respondents are in rural areas, accounting for nearly 70% of the total population. Although, the vast majority of respondents have medical insurance, most of them are the new rural cooperative medical insurance. The level of economic support for the elderly is also relatively low, with an overall average of only 13,300 RMB, of which the eastern region is the highest, with economic support of 16,700 RMB per capita. There is a consequential difference between the eastern regions and the other two regions. The economic support per capita in central and western china is 12,800 and 10,400 RMB, respectively. The differences in economic support among the elderly patients with chronic diseases in the three regions also reflect the regional differences in China's economic development.

**Table 2:** Descriptive Statistics.

	**ALL**	**East**	**Central**	**West**
	**(*N* = 3,416)**	**(*N* = 1,107)**	**(*N* = 1307)**	**(*N* = 1,002)**
**Ecosupport (tk, year)**				
Socioeconomic support	0.99	0.74	0.95	0.65
Family economic support	0.34	0.30	0.33	0.39
**Proportion (%)**				
**Gender**				
Male	21.49	21.77	21.96	20.56
Female	78.51	78.23	78.04	79.44
**Age**				
60–70	52.08	54.47	54.17	46.71
70–80	34.22	31.17	33.21	38.92
Above	13.7	14.36	12.62	14.37
**Marital status**				
Single	24.62	22.58	23.26	28.64
Married	75.38	77.42	76.74	71.36
**Education**				
Primary school or below	76.84	74.25	73.91	85.13
Junior high school	13.47	14.45	15.68	9.48
Senior high school	7.99	8.85	8.49	6.39
College or above	1.7	2.44	1.91	0.6
**Residence**				
The center of city/town	23.45	22.94	25.4	21.46
Combination zone between urban and rural areas	8.05	6.68	10.02	6.99
Village	68.5	70.37	64.58	21.46
**Types of medical insurance**				
Urban employee medical insurance	16.19	12.48	17.52	12.48
Urban and rural resident medical insurance	12.41	11.38	8.57	11.38
Urban insurance medical insurance	5.04	4.29	7.12	4.29
New rural cooperative medical insurance	60.6	65.77	62.28	65.77
Other	5.77	6.96	4.51	61

Besides, we also find that the number of elderly patients with chronic diseases who have earned income of reemployment is few, the amount of earned income of reemployment per capita is less than the pension per capita. According to previous research, many elderly people in China share the responsibility of taking care of their grandchildren, which left limited time for them to work (35, 36). Meanwhile, elderly patients with chronic diseases are limited by their age and health status and these unfavoured characteristic restricted their choices (37). Therefore, pension is the main source of socio-economic support for elderly patients with chronic diseases.

**Figure 1:**
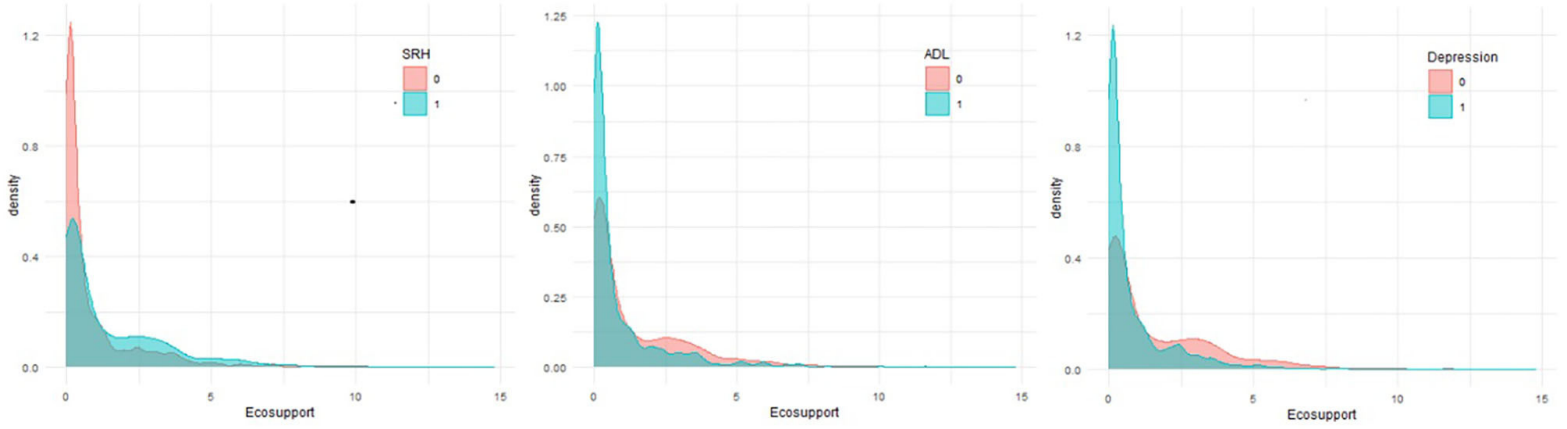
Economic support for different health status.

We visualize the distribution of economic support for elderly patients with chronic diseases. The economic support distribution of elderly patients with chronic disease with different health status under three health dimensions is compared, and a density curve (Figure 1) is drawn on that basis. In order to show the differences of health level intuitively, we unify health level into dichotomous variables. “0” is unhealthy, “1” is healthy. The ordinate represents the distribution density of economic support, whereas, the abscissa depict the amount of economic support. Generally, people with different health condition are categorized into blue and red. In the self-rated health dimension, red describes people with poor health, and blue refered to people with relatively good health. Since mental health and ADL adopt inverse indicators, the color represents the opposite of self-rated health. According to the figure, it could be found that most of elderly patients with chornic disease received <20,000 economic support, the overall economic support level is low. Regardless the health dimension, elderly patients with chronic disease with poor health condition receive less economic support than those with better health. As shown in the figure, although the peak density is in a states of low economic support, the kurtosis of people with poor health is significantly higher than that of people with better health. Therefore, the above analysis suggests that most of the elderly people with chronic diseases with poor health have fairly low levels of economic support. Likewise, the result from the picture also appears to reinforce our conjecture that economic support will affect the condition of elderly patients with chronic diseases.

### The Impact of Economic Support on the Health of Elderly Patients With Chronic Diseases

Table 3 shows the results of the stepwise regression of the influence of economic support on self-rated health. Key independent variables, demographic factors, unhealthy lifestyles (drinking and smoking), physical activities, and medical insurance-related data were included in the model. The results of the four models all show that socio-economic support was beneficial to improve the self-rated health of elderly patients with chronic diseases, and family economic support had no significant impact on self-rated health, indicating that the model was robust. Model 1 shows that without any control variables, socio-economic support has a significant positive impact on self-rated health in elderly patients with chronic diseases. As a component of economic support, both socioeconomic support, and family economic support can improve the self-rated health of elderly patients with chronic diseases, but family economic support fails to pass the significance test. In model 2, model 3 and model 4, demographic indicators, unhealthy lifestyles (drinking and smoking), physical activities, and data related to medical insurance were successively added as control factors. Socioeconomic support still had a significant positive impact on self-rated health of elderly patients with chronic diseases, but the impact was gradually reduced.

**Table 3:** SRH stepwise regression analysis.

**SRH**	**Model 1**	**Model 2**	**Model 3**	**Model 4**
Socioeconomic support	0.1848^***^	0.1092^***^	0.0994^***^	0.0783^***^
	−0.0188	−0.0237	−0.0243	−0.0238
Family economic support	0.0400	0.0383	0.0393	0.0261
	−0.0437	−0.0438	−0.0438	−0.0435
Gender		−0.1323	−0.1305	−0.1748
		−0.0868	−0.0867	−0.1011
Age		−0.0066	−0.0069	−0.0014
		−0.0052	−0.0052	−0.0053
Marital status		0.1304	0.1255	0.1036
		−0.0849	−0.0850	−0.0853
Education		0.1554^***^	0.1499^***^	0.1282^**^
		−0.0577	−0.0577	−0.0577
Residence		0.1559^***^	0.1292^***^	0.1425^**^
		−0.0470	−0.0492	−0.0493
Types of medical insurance			−0.0636^**^	−0.0676^**^
			−0.0320	−0.0320
Reimbursement			−0.0108^**^	−0.0088^***^
			−0.0047	−0.0042
Smoke				−0.3248^**^
				−0.1417
Activity				0.3272^***^
				−0.0501
Drink				−0.2165^***^
				−0.0536

Standard errors in parentheses

* *p < 0.1*,

** *p < 0.05*,

*** *p < 0.01*.

Since mental health and activity of daily life are different from self-rated health indicators, the self-rated health value 0 means unhealthy. Mental health and activity of daily life value 0 means healthy. Therefore, the regression results of mental health status and activity of daily life are presented in Table 4. Without any control variables, socioeconomic support had a significant positive effect on both. Similar to self-rated health, family economic support had no significant impact on mental health and activity of daily life. As the control variables included in sequence, the influence of socioeconomic support gradually decreased. When all the control variables were included, the influence of socioeconomic support on activity of daily life was no longer significant, and mental health status was still significant.

**Table 4:** Mental health/ADL stepwise regression analysis.

	**Model 1**		**Model 2**		**Model 3**		**Model 4**	
	**Depression**	**ADL**	**Depression**	**ADL**	**Depression**	**ADL**	**Depression**	**ADL**
Socioeconomic support	−0.3432^***^	−0.1976^***^	−0.1780^***^	−0.1068^***^	−0.1613^***^	−0.0803^**^	−0.1537^***^	−0.0517
	−0.0268	−0.0269	−0.0324	−0.0342	−0.0341	−0.0349	−0.0342	−0.0341
Family economic support	−0.0463	−0.0094	−0.0433	−0.0196	−0.0439	−0.0333	−0.0419	−0.0217
	−0.0437	−0.0047	−0.0448	−0.0051	−0.0448	−0.0051	−0.0448	−0.0052
Gender			0.2259^**^	0.2534^**^	0.2279^**^	0.2517^**^	0.2209^**^	0.3203^***^
			−0.0895	−0.1008	−0.0895	−0.1013	−0.1049	−0.1203
Age			−0.0050	0.0451^***^	−0.0047	0.0460^***^	−0.0075	0.0351^***^
			−0.0052	−0.0056	−0.0052	−0.0056	−0.0053	−0.0058
Marital Status			−0.1649^*^	−0.3593^***^	−0.1602^*^	−0.3424^***^	−0.1512^*^	−0.3106^***^
			−0.0851	−0.0906	−0.0852	−0.0911	−0.0853	−0.0932
Education			−0.3289^***^	−0.2181^***^	−0.3263^***^	−0.2021^***^	−0.3201^***^	−0.1783^**^
			−0.0663	−0.0728	−0.0663	−0.0731	−0.0663	−0.0737
Residence			−0.2600^***^	−0.1450^***^	−0.2434^***^	−0.1029^*^	−0.2457^***^	−0.1096^*^
			−0.0490	−0.054	−0.0504	−0.0564	−0.0505	−0.0572
Types of medical insurance					0.0510	0.1253^***^	0.0521	0.1307^***^
					−0.0350	−0.0379	−0.0350	−0.0385
Reimbursement					−0.0018	0.0236^***^	−0.0013	0.0201^***^
					−0.0036	−0.0061	−0.0036	−0.0061
Smoke							0.0190	0.1652
							−0.1425	−0.1616
Activity							−0.1313^***^	−0.5932^***^
							−0.0493	−0.056
Drink							0.0566	0.1386^**^
							−0.0555	−0.0663

Standard errors in parentheses

* *p < 0.1*,

** *p < 0.05*,

*** *p < 0.01*.

### Regional Differences in the Impact of Economic Support on Health

Due to the uneven development among different regions in China, this paper divides the eastern, central, and western regions of China and makes regression analysis on their health status, respectively to study the difference of the impact of economic support on health among different regions. The regression results are shown in Table 5.

**Table 5:** Regional differences in economic support.

	**West**			**Central**			**East**		
	**SRH**	**Depression**	**ADL**	**SRH**	**Depression**	**ADL**	**SRH**	**Depression**	**ADL**
Socioeconomic support	0.1754^***^	−0.0745	0.0726	0.0411	−0.1965^***^	0.0136	0.0670^*^	−0.1600^***^	−0.1789^***^
	−0.0657	−0.0709	−0.0791	−0.0441	−0.0652	−0.0388	−0.0308	−0.0509	−0.061
Family economic support	0.0088	0.0497	−0.0143	0.0583	0.0314	0.0521	0.076	−0.0979	0.0177
	−0.0094	−0.0939	−0.0119	−0.0793	−0.0859	−0.0091	−0.0631	−0.0676	−0.0076
Gender	−0.0507	0.5145^***^	0.1705	−0.1978	0.2798^*^	0.3302^*^	−0.2819	−0.0938	0.4099^*^
	−0.1865	−0.1943	−0.2238	−0.1614	−0.1667	−0.1844	−0.1819	−0.1918	−0.2325
Age	0.0153	−0.0073	0.0380^***^	−0.0094	−0.0055	0.0279^***^	−0.0047	−0.0138	0.0437^***^
	−0.0099	−0.0099	−0.0109	−0.0087	−0.0087	−0.0093	−0.0093	−0.0097	−0.0105
Marital status	0.1832	0.0539	−0.3203^*^	0.1227	−0.2110	−0.3445^**^	0.0251	−0.1997	−0.2187
	−0.1530	−0.1503	−0.1686	−0.1411	−0.1421	−0.1531	−0.1559	−0.1575	−0.1765
Education	0.1864	−0.4133^***^	−0.3561^**^	0.1430	−0.3292^***^	−0.2426^**^	0.0690	−0.2333^***^	0.0064
	−0.1274	−0.1381	−0.1699	−0.0902	−0.1051	−0.1106	−0.0949	−0.1125	−0.129
Residence	0.1244	−0.3652^***^	−0.2039^*^	0.1911^**^	−0.2328^***^	−0.118	0.1196	−0.2191^***^	−0.0476
	−0.0985	−0.0954	−0.1141	−0.0783	−0.0799	−0.0873	−0.0859	−0.0959	−0.1094
Types of medical insurance	0.0523	0.0684	0.2504^***^	−0.1281^**^	−0.0171	0.1396^**^	−0.055	0.0671	0.0111
	−0.0674	−0.0681	−0.0809	−0.0554	−0.0629	−0.064	−0.0509	−0.0566	−0.0659
Reimbursement	−0.0075	−0.0040	0.0321^**^	−0.0648^***^	0.0106	0.0288^**^	−0.0055^***^	0.0017	0.0136^*^
	−0.0118	−0.0106	−0.0131	−0.0159	−0.0120	−0.0131	−0.0036	−0.0041	−0.0082
Smoke	−0.6170^**^	0.3891	0.0634	−0.1074	0.0329	0.2014	−0.3239	−0.3178	0.174
	−0.2751	−0.2684	−0.3146	−0.2207	−0.2241	−0.2414	−0.2551	−0.2679	−0.3131
Activity	0.0884	0.0299	−0.4772^***^	0.4175^***^	−0.3081^***^	−0.6338^***^	0.4700^***^	−0.3178^*^	−0.6586^***^
	−0.0971	−0.0934	−0.1068	−0.0821	−0.0819	−0.0911	−0.0868	−0.0858	−0.1008
Drink	−0.3901^***^	0.0791	0.2723^**^	−0.1027	0.0057	0.0215	−0.1838^*^	0.0979	0.1817
	−0.988	−0.0970	−0.1225	−0.0869	−0.0914	−0.1025	−0.0964	−0.1036	−0.1298

Standard errors in parentheses

* *p < 0.1*,

** *p < 0.05*,

*** *p < 0.01*.

The results show that the influence of socioeconomic support on the health of elderly patients with chronic diseases is significantly different in regions with different levels of economic development, and the influence of family economic support on the health of elderly patients with chronic diseases is still not significant. In all regions, socioeconomic support had a significant impact on self-rated health. On the contrary, the activity of daily life, socioeconomic support only has an impact in developed regions, but not in undeveloped and less developed regions. In mental health, socioeconomic support has no effect on mental health in undeveloped areas but has a significant positive effect on central and western china. Thus, it might be suggested that the impact of economic support on health is affected by the economic development degree of the region. Economic supports in different regions have different impact on all aspects of health.

## Discussion

Based on CHARLS database, this paper studies the impact of economic support on the health of elderly patients with chronic diseases. Both stepwise regression and regional regression show that socioeconomic support have a significant impact on the health status of elderly patients with chronic diseases, which is basically the same as the previous research results of scholars (38–40).

Socioeconomic support can effectively improve the health status of elderly patients with chronic diseases, especially in terms of self-rated health and mental health. Since the daily life ability is greatly affected by individual unhealthy lifestyles and physical activities, the influence of socioeconomic support on daily life ability decreases after the inclusion of health behavior.

The condition of individual economy is an important factor affecting health, especially for the elderly. Andrews argues that higher levels of retirement income or wealth leads to increased general well-being, which improves the health of the elderly (41). According to Maslow's hierarchy of needs, the individuals will pursue further needs only when the basic survival need is fulfilled (42). The research of Zacharias Dermatis finds that the elderly with higher annual incomes have better quality of life in all aspects than the elderly with lower annual incomes (43). With a stable economic source, the elderly are more willing to pay attention to the improvement of their health condition. Patients with chronic diseases have a high degree of medicial treatment dependence, and the economic burden of drugs is significantly higher than that of other groups (44). Due to the lack of income sources and the high economic burden of the elderly, the level of economic support determines the quality of life of the elderly with chronic diseases. Elderly patients with chronic diseases with low economic support lack sufficient funds to maintain their own health status, and the deterioration of their own health will further aggravate the economic burden of drugs, forming a vicious circle. The deterioration of health status will also bring about a series of chain reactions. Elderly patients with chronic diseases suffer from chronic diseases for years, bringing psychological trauma. The degree of economic support can effectively alleviate the economic concerns of patients, so that they can face the diseases with a more relaxed emotion and attitude and then improve their psychological conditions.

Compared with socioeconomic support, the intervention effect of family economic support on health is not significant. Family economic support has no significant impact on health, whether it was self-rated health, mental health, or ability to live a daily life. This is because family economic support is significantly lower than that of socioeconomic support. Although, the research of Xue Long and some other scholars believed that the economic support has an impact on health status, the *P*-value in their research is too large to be convinced as strongly significant (45). Children's economic support for their elders usually only ensures normal life needs but fails to meet their elders' deeper health needs (46). For the elderly chronic disease group with pension, family economic support is obviously insufficient in terms of quantity and stability compared with socioeconomic support, which has less impact on health. Elderly people with chronic diseases without adequate socioeconomic support are more likely to live in poor family economic conditions, and their children might unable to provide adequate economic security to maintain the health of patients. In addition, the rural population of CHARLS database is significantly higher than that of the urban population, accounting for about 70%. The higher rural sample proportion might lead to low family economic support, resulting in the overall low family economic support (47). In China, elder has a strong tradition mindset of the necessity of sufficient bank saving. They are less willing to invest in health consumption. When the economic support is low, the elderly with chronic diseases are more willing to keep their current assets like cash for the rainy day rather than investing in health (48). Therefore, there may be a counteractive effect of family economic support on health status. It can be explained that family economic support, compared with socioeconomic support, cannot play an effective role in improving the health status of elderly patients with chronic diseases.

As the economic support is limited by the degree of local economic development, this paper divides China into three parts according to the level of economic development and geographical factors, corresponding to undeveloped regions, less developed regions and developed regions in turn. The economic condition of the eastern region is significantly higher than that of the central and western regions (49). The level of economic support and health status of elderly patients with chronic diseases is also significantly higher than that of the other two regions. Family economic support in the three regions has no significant impact on health, and there are clear regional differences in the impact of socioeconomic support on health status.

Socioeconomic support cannot play a role in the self-rated health of the elderly patients with chronic diseases in the central regions, but significantly improve the health status of the elderly patients with chronic diseases in the western regions. Evans, MC study finds that economic difficulties have an impact on the health of low-income elderly persons and suggested that solving economic difficulties may help to promote the health of the elderly (50). The interpretation of this paper is that in undeveloped regions, the family economic condition cannot meet the normal health needs of elderly patients with chronic diseases, thus, worsening the health status of the elderly. Therefore, the socioeconomic support in the western region can effectively improve the self-rated health of the elderly. The socioeconomic support can guarantee the primary expenditure of elderly patients with chronic diseases and maintain the health status of elderly patients with chronic diseases. In the eastern region, the social and economic support has an impact on self-rated health, but the *P*-value is close to 0.1. The influence of economic support on self-rated health is far less than that in western China. When primary health needs are met, elderly patients with chronic diseases will have higher level of health needs, and psychological needs begin to appear gradually (51). Meanwhile, Gao et al. (52) find in their study that, under the influence of traditional Chinese family values, the elderly would also provide certain economic support to their children. Elderly patients with chronic diseases in the central region have a greater burden of economic support for their children, and the impact of socioeconomic support on mental health is more obvious than that in the eastern region. Unlike the results of stepwise regression, the socioeconomic support of the elderly patients with chronic diseases in the eastern region can effectively improve their activity of daily life. Due to the relatively developed economy in the eastern region, the elderly patients with chronic disease receive more socioeconomic support, and they are more willing to choose treatment or recuperation when there are physical abnormalities. The elderly patients with chronic diseases in the central and western regions are limited by economic pressure, and seldom pay attention to them.

Compared with previous studies, this study has some innovation. Firstly, this paper discusses the influence of economic support on health status from two dimensions: social and family. Previous studies mostly used single economic indicators, such as, pensions and family economic support, to study their impact on health, without taking into account the multidimensionality of economic support for the elderly (22, 23). Secondly, the measurement of health status in this paper reflects the health status of elderly patients with chronic diseases more comprehensively from the self-rated of health and mental health based on the subjective judgment of respondents and the daily life ability of objective judgment, and explores the impact of economic support on health under different health dimensions. The third, this paper divides China into different regions according to the level of regional economic development and geographical location, and further discusses the heterogeneity of the influence of economic support between different regions on the health status of elderly patients with chronic diseases, which is conducive to the formulation and improvement of regional policies.

Our study also had some limitations. First of all, we used cross-sectional data. The research results can only reflect the impact of economic support on the health status of elderly patients with chronic diseases but cannot explain the causal relationship between the two. Secondly, we did not distinguish specific chronic diseases and studied the difference in the impact of economic support among different chronic diseases. Third, due to the limitation of CHARLS database, there are a large number of rural samples, which may lead to a lower level of economic support.

## Conclusion

By analyzing the results of the study, we conclude that economic support has a significant positive impact on the health of elderly patients with chronic diseases. The government should pay special attention to the socioeconomic support for elderly patients with chronic diseases and formulate some policy that can improve the economic support for elderly patients with chronic diseases, such as, appropriately increasing the pension amount, expanding the scope of assistance for serious diseases and the scope of medical insurance reimbursement. In this paper socioeconomic support is composed of pension and earned income of reemployment, while the number of respondents who has earned income of reemployment is few. Therefore, increasing socio-economic support is mainly through increasing pension amount. These are key methods to improve the health status of elderly patients with chronic diseases. At the same time, we also find that there are huge regional differences in the impact of economic support on health. It is the long-term goal of the Chinese government to continuously reduce the economic differences among regions to improve the health imbalance of the elderly patients with chronic diseases among regions.

Tragically, large numbers of elderly patients with chronic diseases at the bottom are unable to get enough economic support to keep healthy. Many countries are still trying to take measures to deal with the health problems of elderly patients with chronic diseases (53, 54). The results of this paper suggest that economic support could improve the health status of elderly patients with chronic diseases. The communities and regions facing the triggers of the health state of elderly patients with chronic diseases could consider increasing economic support as a part of the policy. However, according to Daroudi et al. (55), the economic cost of improving health varies from country to country at different economic levels. Therefore, before deciding whether to increase social support to improve health, different countries need to conduct an accurate assessment of their level of development to make sure that improved social support has the same effect. It is worth noting that due to differences in the national conditions and cultural backgrounds, when formulating policies to increase economic support, other countries can consider which aspect to increase economic support for elderly patients with chronic diseases.

## Data Availability Statement

Publicly available datasets were analyzed in this study. This data can be found here: https://charls.pku.edu.cn.

## Author Contributions

Study conception and design were performed by ST. Data analysis was performed by TY and DQ. The first draft of the manuscript was written by YX. ZL checked the data and edited the language. All authors commented on previous versions of the manuscript, contributed to the material preparation and data collection, read, and approved the final manuscript.

## Conflict of Interest

The authors declare that the research was conducted in the absence of any commercial or financial relationships that could be construed as a potential conflict of interest.
